# Does total hip arthroplasty result in intercostal nerve injury? A case report and literature review

**DOI:** 10.1515/med-2023-0731

**Published:** 2023-06-05

**Authors:** Zhengming Wang, Jingjing Zhang, Rui Wang, Quanquan Chen, Peijian Tong, Hongsheng Zhan, Shuaijie Lv

**Affiliations:** Shuguang Hospital Affiliated to Shanghai University of Traditional Chinese Medicine, Shanghai, China; The First Affiliated Hospital of Zhejiang Chinese Medical University (Zhejiang Provincial Hospital of Chinese Medicine), Zhejiang, 310006, China; The First Affiliated Hospital of Zhejiang Chinese Medical University (Zhejiang Provincial Hospital of Chinese Medicine), 54 Youdian Road, Hangzhou, Zhejiang, 310006, China

**Keywords:** neuropathy, intercostal nerve, position, total hip arthroplasty

## Abstract

Neuropathy in surgical-related locations has received concerns after total hip arthroplasty (THA), while the contralateral intercostal nerve (ICN) injury has not been reported. A 25-year-old female patient with a body mass index (BMI) of 17.9 kg/m^2^ visited the orthopedic outpatient clinic with complaints of progressive left hip pain for 20 days. She was diagnosed with left end-stage hip osteoarthritis and developmental dysplasia of the bilateral hips after radiographs and a detailed history-taking. After painstaking consideration, a cementless THA with the standard posterolateral approach was performed under general anesthesia. The procedure was difficult but successful. Unexpectedly, the numbness and slight tingling in the skin of the right breast, lateral chest wall, and axilla cropped up on the first postoperative day. Following the clinical features and the conclusion of the multidisciplinary discussion, we assume that ICN neuropathy is the diagnosis in this case due to compression of the lateral decubitus position during the operation. Her symptoms completely disappeared after using mecobalamin injection (0.5 mg, intramuscular injection, every other day) for 11 days. The Harris left hip score improved from 39 to 94, and the visual analogue scale from 7 was reduced to 2 on the day of discharge. There were no other complications within the first year after the operation. For THA, we should pay attention to some unexpected complications by virtue of the special position, especially in thin and low-BMI people, which suggested that further comprehensive perioperative nursing measures and the beneficial surgical position and anesthesia type were called for.

## 1 Introduction

For its astonishing efficacy, total hip arthroplasty (THA) has been widely used and has a long history dating back to the 1960s [1,2,3]. Notably, THA remains one of the most successful orthopedic interventions currently, with a survival rate of 95% at 10 years postoperatively [4]. Owing to the increasing number of THA candidates, the procedure is expected to increase to 635,000 procedures annually by 2030 [5]. The postoperative complications of THA, although fully emphasized and prevented as much as possible, are still an alarming problem, mainly including thrombosis, infection, malunited incision, etc., which reduce patient satisfaction, increase the cost of medical insurance, and extend the length of stay (LOS) [6,7,8,9]. This article presents a case of intercostal nerve (ICN) injury caused by the THA through a standard posterolateral approach in the lateral decubitus position under general anesthesia (GA). This observation aimed to highlight the differences in muscle or fat between thin or low body mass index (BMI) people and others, in this manner appealing to the beneficial surgical position and the anesthesia type for someone special.

## 2 Case presentation

**Figure 1:**
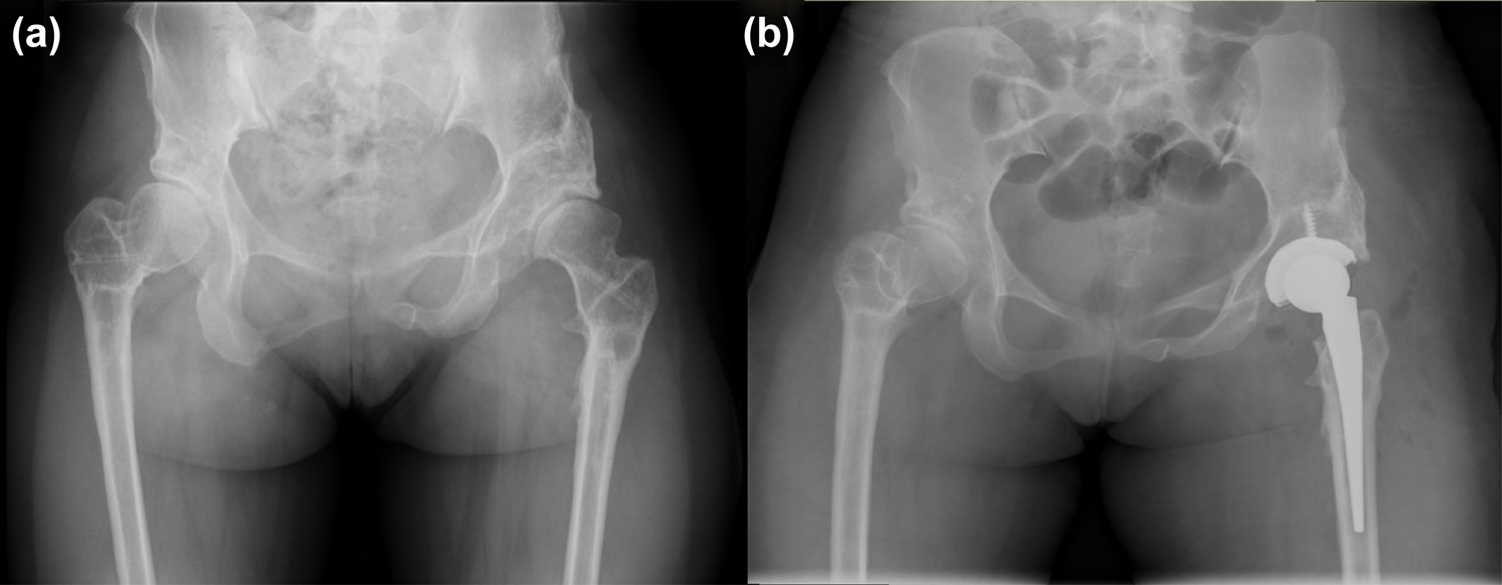
The X-rays of the case. (a) Preoperative plain radiography of the pelvis. (b) Postoperative plain radiography of the pelvis.

A 25-year-old female patient with a BMI of 17.9 kg/m^2^ visited the orthopedic outpatient clinic complaining of progressive left hip pain for 20 days. A detailed history-taking revealed no incentive to develop bilateral hip pain in her adolescence. She went to the local hospital and was diagnosed with bilateral hip dislocation and developmental dysplasia of the hip. With her informed consent, open reduction, Dega osteotomy, and proximal femoral shortening combined with derotation osteotomy were performed. She recovered well from the operation and had no symptoms until she overstepped due to tourism last month. After getting home, she felt severe pain in her left hip but not on the right side. The patient denied any other previous medical history. She had no family history of infectious diseases, inflammatory arthritis, or malignancy. On physical examination, her left hip had obvious local tenderness, with limited range of motion, with 12 degrees of abduction, 10 degrees of adduction, 70 degrees of flexion, and 10 degrees of posterior extension. The left Harris Hip Score (HHS) was 39, and the visual analogue scale (VAS) was 7. Further physical examination revealed positive FABER tests, log roll tests, and flexion-internal rotation-adduction impingement tests on the left hip. Radiographs revealed advanced left hip osteoarthritis (Figure 1a). Combining the patient’s willingness to undergo surgery with the situation, we proposed a THA on her left hip. With this surgical plan, a written informed consent was obtained.

**Figure 2:**
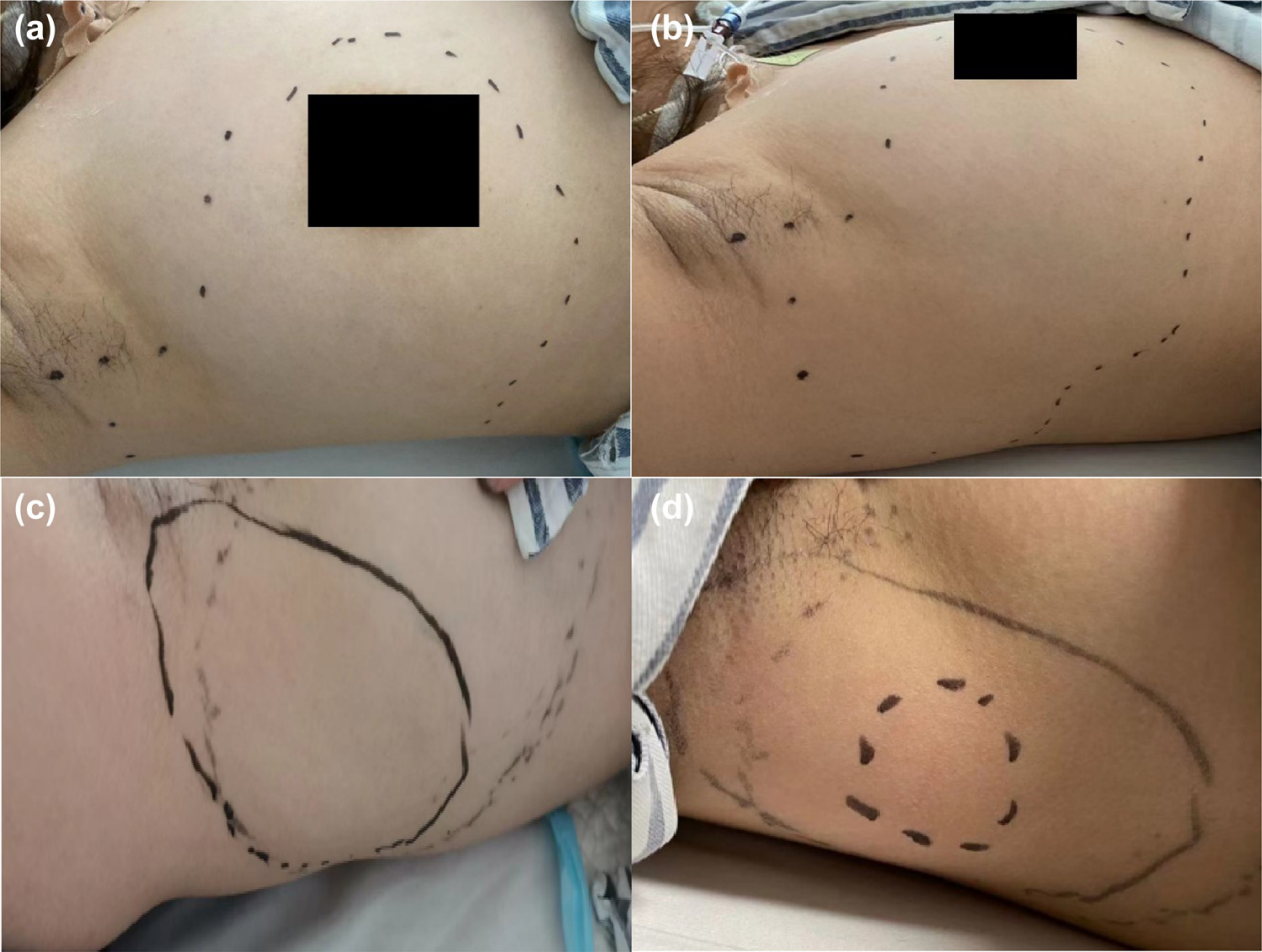
The postoperative paresthesia location of the case. (a–b) The dotted line circle indicated the paresthesia location on the first day after the operation. (c) The dotted line circle indicated the paresthesia location on the fifth day after the operation. (d) The dotted line in the circle indicated the location of the paresthesia on the ninth day after the operation.

After adequate preoperative preparation, the left hip was treated with a cementless THA through a standard posterolateral approach under GA. The surgical procedure was uneventful and took only 87 min. The amount of blood loss intraoperatively was 80 mL. After surgery, 1.5 g IV drops of cefuroxime were used to prevent infection with low-molecular-weight heparin anticoagulation. Postoperative plain radiographs showed that total hip components were in good position and aligned (Figure 1b). She told us that there was numbness and slight tingling in the skin of the right breast, lateral chest wall, and axilla (Figure 2a and b) on the first postoperative day, just as we were glad for her successful operation. As a result of the complication, she experienced a depressed mood and doubted the rationality of the operation.

This was given a lot of weight, and in order to identify the inducement, we analyzed the entire course of treatment. The imaging tests and laboratory analyses were performed, including a breast ultrasound, an X-ray of the hip, an ICN electromyogram, a C-reactive protein test, an erythrocyte sedimentation test, and a tumor serum analysis, all of which were negative. The patient has no breast problems and no visible psychiatric abnormalities, according to a detailed conclusion reached after a multidisciplinary consultation with a breast surgeon, a neurologist, a psychologist, and an anesthesiologist. Further, these symptoms were similar to those when the ICNs were squeezed and pulled after breast surgery. Given the urgency of repairing a nerve injury, the symptomatic treatment was performed immediately. The mecobalamin injection (0.5 mg, intramuscular injection, every other day) and psychological counseling were started to accelerate recovery in tandem with the above.

**Figure 3:**
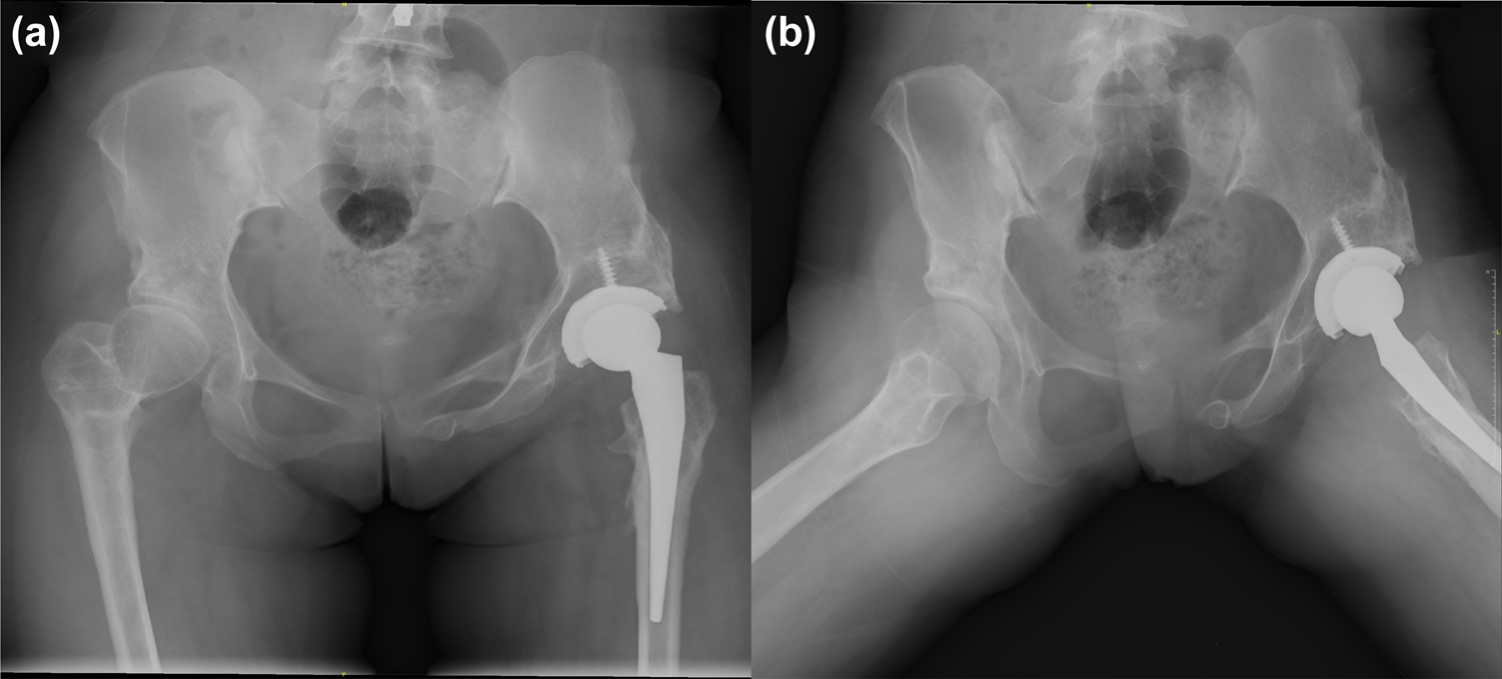
The X-rays of the case in the postoperative 3 months. (a) The anteroposterior radiography. (b) The frog-leg lateral radiography.


The symptoms improved with time (Figure 2c). Only a minor portion of her lesion remained after 9 days (Figure 2d). Fortunately, there were no other complications during the hospitalization. The VAS dropped from 7 to 2, while the left HHS increased from 39 to 94. She was joyfully discharged on the 12th postoperative day owing to vanishing discomfort. During the follow-up, none of the aforementioned symptoms returned. Figure 3 displays the X-rays taken 3 months following the procedure. Throughout the first year following the procedure, there were no complications.

**Ethical review committee statement:** This study was approved by the Ethics Committee of the First Affiliated Hospital of Zhejiang Chinese Medical University and obtained permissions to access clinical/personal patient data used in our research. Written informed consent was obtained from the participant in the study. And a written informed consent was obtained from the patient to publish this study and any accompanying images.
**Consent for publication:** The patient declared that she agree to publish.

## 3 Discussion

There are many criteria for evaluating the success of a THA, including the amount of intra- and postoperative blood loss, the appropriate placement of the prosthesis, the amelioration of joint function and pain, and so on; however, the most important may be the appearance of the postoperative complication [10]. For this patient, many complications, such as infection, deep vein thrombosis, and prosthesis dislocation, were effectively prevented through the collectively collaborative endeavor. Unexpectedly, the paraesthesia of the breast, lateral chest wall, and axilla, which is unquestionably a rather complex multifactorial syndrome, manifested at the contralateral surgery site. We assume that ICN neuropathy is the diagnosis in this instance based on the clinical characteristics and the outcome of the multidisciplinary discussion.

Twelve ICNs supply the sensory innervation for much of the back, trunk, upper abdomen, and intercostal muscles, each originating from spinal nerve roots at the same vertebral level as the rib they travel with. Near the midaxillary line, the ICN sends an offshoot called the lateral cutaneous branch, which travels laterally through the internal and external intercostal muscles and then divides into a dorsal and ventral branch. As before, the anterior cutaneous branch, which splits into a lateral and medial branch, is sent when the individual ICNs cease [11]. The lateral cutaneous branches give cutaneous information from the skin of the lateral thoracic wall. In contrast, the anterior cutaneous branches are the terminal branches of the ICN, which supply the skin of the anterior thoracic wall [12]. Intercostal neuropathies are a serious and common side effect caused by various thoracic procedures and antineoplastic agents, which present predominantly as sensory axonal neuropathy with considerable variability of clinical symptoms [13]. In crush injuries, which can potentially have life-threatening systemic consequences, neuropathy is also frequent [14,15,16]. In this case, the patient suffered from an ICN injury predominantly brought on by lateral decubitus position compression, which can have a debilitating effect, trigger off symptoms of anxiety and depression, and reduce health-related quality of life. Moreover, neuropathy, as we all know, is remarkably arduous to deal with and takes a lot of time to repair. Consequently, it is crucial to avoid its emergence.

There were a few possible causes of postoperative non-operative site neurapraxia in this patient. First, the patient was exposed to compression neuropathies in the fixed lateral decubitus position during THA. Second, the GA results in the absence of the typical defensive reactions to paresthesias and other indications of nerve damage [17]. Low muscular tone contributes to the compression of ICNs. In addition, injuries may be made worse by the anesthetic agent’s direct toxic action on peripheral nerves [18,19]. Finally, the subcutaneous tissue may thin out and become less effective in defending the nerves due to the low BMI. Based on the above, we achieved some enlightenment.

To begin with, for patients with a smaller BMI or who are thinner, the supine position may be more preferred in THA, and specific care is essential. Undoubtedly, the appropriate position is a significant factor in carrying out the procedure smoothly, and the approach and surgical position are strongly connected. The posterolateral approach or direct anterior approach (DAA) is commonly used in traditional THA. The former mainly adopts a lateral decubitus position, while the latter can choose a supine or lateral position. Specifically, both the lateral decubitus position and the supine position have benefits and drawbacks; which of the two positions is preferable still remains controversial. Performing THA in the supine position has an anesthesiological advantage and more accuracy in the implantation of the acetabular cup, but it has a longer operation time, a longer incision length, and more blood loss and transfusions in overweight and obese patients compared with the lateral decubitus position [20,21]. When a THA with the supine DAA was performed for the fixed limb on a specific operating table, the capacity to undertake dynamic testing (the push–pull test) was eliminated [22]. However, the majority of posterior approach surgeons undertake dynamic evaluations on the hip stability and the soft tissue tension during the procedure, which is predicted prosthesis stability after operation [23]. In addition, because the pelvic position was fixed in the supine DAA and both lower limbs could be disinfected simultaneously, the lengths of both lower limbs could be compared, which led to a relatively high accuracy in the postoperative absolute length of the lower extremity [24]. Moreover, a larger error in inclination and anteversion was observed with the supine position, using the muscle-sparing modified Watson-Jones approach [25]. However, the impact of the surgical position on ICN palsies has received little investigation. We hold that the intensity in the lateral position is higher in thin people, which may bring about inferior outcomes. More high-quality research should be performed to determine the clinical impact of the operative position.

Besides, although multiple studies showed evidence of decreased postoperative complications and LOS with regional anesthesia (RA), there is no eventual conclusion that THA was performed with it because there is no clear evidence of the superiority of RA over GA with respect to mortality [26,27,28,29,30]. Welch et al. [31] reported that the use of GA or epidural anesthesia increased the risk of postoperative neuropathy, but there was no difference with the use of peripheral nerve blockade. It is a controversial proposition about whether to use GA or RA for THA. As there were normal protective reactions to indications of peripheral neuropathy in nonsurgical operative regions during a THA with RA, we surmised that RA should be preferred for patients without accompanying comorbidities in the future.

## 4 Conclusion

It is necessary to broaden the evaluation scope of the success of THA and postoperative patient satisfaction, which is not only limited to postoperative infection and pain but also affects nonoperational locations. For THA, we should pay attention to some unexpected complications by virtue of the special position, especially in thin and low-BMI people, which suggested that further comprehensive perioperative nursing measures and the beneficial surgical position and anesthesia type were called for.

## Abbreviations

THA: total hip arthroplasty
LOS: length of stay
ICNs: intercostal nerves
BMI: body mass index
DDH: developmental dysplasia hip
ROM: range of motion
HHS: Harris Hip Score
VAS: visual analogue scale
DAA: direct anterior approach
GA: general anesthesia
RA: regional anesthesia
